# Collagen-Based Bioengineered Substitutes of Donor Corneal Allograft Implantation: Assessment and Hypotheses

**Published:** 2012

**Authors:** Nataliya Pasyechnikova, Volodymyr Vit, Mykola Leus, Stanislav Iakymenko, Oleksiy Buznyk, Sergii Kolomiichuk, Illia Nasinnyk, Mohammad Mirazul Islam, May Griffith

**Affiliations:** 1 The Filatov Institute of Eye Diseases and Tissue Therapy, Odessa, Ukraine,; 2 Integrative Regenerative Medicine Centre, Linköping University, Linköping, Sweden

**Keywords:** Bioengineered substitutes of corneal stroma, Cross-linking collagen, Ukraine

## Abstract

To fabricate donor corneal substitutes based on carbodiimide cross-linked porcine collagen, to study their in vitro and in vivo properties, and to elaborate new implantation techniques for the donor corneal collagen-based substitutes, this study had been performed. Bioengineered substitutes of corneal stroma (BSCS) were fabricated by cross-linking porcine type I collagen with 1-ethyl-3-(3-dimethyl aminopropyl) carbodiimide and N-hydroxysuccinimide, as previously described. Their refractive indices were measured using an Abbe refractometer. The mechanical properties were evaluated by their ability to tolerate interrupted stitches placed during deep lamellar keratoplasty performed on isolated rabbit eyes. BSCS were then implanted into one cornea of 8 rabbits and were followed-up for 12 months. Our BSCS had refractive indices of 1.24-1.3 (human cornea 1.37-1.38), and tolerated the placement of 12 interrupted stitches well. A new technique, the BSCS “stitchless” implantation, was developed. When implanted into rabbit corneas, BSCS remained stably integrated and clear during the 12 month follow-up. Non-intensive opacities within corneal layers (grade 1.5 on a scale of 0 to 4) were observed in 2/8 eyes during the 1st postoperative week, and in one eye the opacity resolved. In the 2nd eye a fine opacity (grade 1) remained. Light microscopy confirmed the integrity of the implants and the absence of inflammation in corneal stroma. The current data suggest that the BSCS fabricated in the Ukraine by cross-linking collagen is a good alternative to human donor corneas if medical grade porcine collagen is used. In addition, the new “stitchless” technique of BSCS implantation may decrease corneal substitute damage and accelerate its epithelialisation.

## INTRODUCTION

Corneal diseases are a major cause of vision loss, second only to cataracts in overall importance, and affect more than 10 million individuals worldwide [1, 2]. Ocular trauma and corneal ulceration due to infection are significant causes of corneal blindness that are often underreported but may be responsible for 1.5–2.0 million new cases of monocular blindness every year [1]. Currently, the only widely accepted corneal blindness treatment is transplantation of a human donor cornea. The worldwide demand for transplantation corneas exceeds the supply, and this situation will worsen with an aging population and the increased use of cornea laser surgery [3]. In 2010, there were 2000 individuals waiting for corneal transplantation in the Ukraine, but only 357 corneal transplants were performed [4]. 

Alternatives of human donor cornea used in ophthalmology to date include human amniotic membranes [5, 6] and artificial substitutes (keratoprosthesis) [7, 8]. However, the use of amniotic membrane is limited to corneal ulcer treatments as a patch due to its ability to promote healing and it cannot be used for optical purposes. Keratoprostheses are used to restore vision in complicated leukomas. Despite some progress achieved in this area in recent years, the devices have limited indications to their use. Existing prostheses neither integrate seamlessly into the host tissue, nor promote reinnervation. 

Recent studies have shown proof-of-concept for the use of biomimetic materials as corneal implants to promote corneal regeneration as an alternative to donor cornea implantation [9-11]. Implants were fabricated from carbodiimide cross-linked collagen. Their optical, chemical and physical properties were similar to human corneas. When used as corneal substitutes for transplantation, implants stimulated both corneal tissue and nerve regeneration in the experimental animals and patients, without the need for immunosuppression. 

However, these early implants had tensile strengths that were significantly weaker in comparison to the human cornea [10]. That is why the implants were sutured to the recipient cornea with overlying stitches in the majority of cases, but not continuous or separate ones as in conventional corneal transplantation. Overlying sutures may both damage the implant and impede implant coverage with corneal epithelium, as seen in the clinic study [11]. Interpenetrating networks of collagen and phosphorylcholine [12] are more robust, but have yet to be tested clinically.

The purpose of this study was to fabricate donor cornea substitutes based on carbodiimide cross-linked porcine collagen, to study their in vitro and in vivo properties and to elaborate new implantation techniques for the donor cornea collagen-based substitutes to prevent possible implant damage and delay epithelial coverage.

## HYPOTHESES

**Implant fabrication**

Bioengineered substitutes of corneal stroma (BSCS) were fabricated by cross-linking porcine type I atelocollagen (Koken, Japan) with 1-ethyl-3-(3-dimethyl aminopropyl) carbodiimide (Sigma-Aldrich, Canada) and N-hydroxysuccinimide (Sigma-Aldrich, Canada), as previously described [9], collagen concentration in gel was 12%.

**Implant testing in vitro**

Refractive indices of the BSCS were measured using an Abbe refractometer (Bellingham & Stanley, UK). The mechanical properties were evaluated by the ability to tolerate interrupted stitches placed during deep lamellar keratoplasty performed on isolated rabbit eyes.

**Implantation and clinical evaluation**

The study was approved by the Bioethics Committee of the Filatov Institute of Eye Diseases and Tissue Therapy. The animals were operated on, followed-up and sacrificed under the Declaration of Helsinki recommendations for the use of animals in experimental studies. BSCS (6mm in diameter) were implanted into one cornea of 8 New Zealand rabbits with a new “stitchless” technique, and were followed-up for 12 months. Animals were only given antibiotics topically 4 times daily during the first week after the surgery. No immunosupression was used. Stitches were removed at 3 weeks postoperative. Follow-ups were performed daily on each animal for up to 7 days postoperative, and then weekly. Slit-lamp biomicroscopy was used to examine the corneas and the BSCS for optical clarity, as well as to look for any inflammation (as indicated by excessive redness or swelling compared to the un-operated contralateral control eye) or neovascularisation. Other tests included sodium fluorescein staining to assess epithelial integrity and barrier function. 

**Histopathologic Evaluation**

Animals were sacrificed at 12 months post-implantation. Corneas with implants, and control un-operated corneas were processed for histopathological examination by light microscopy after the sections were stained with H&E (Hematoxylin and Eosin stain). 

## RESULTS

**Testing in vitro**

**Figure 1:**
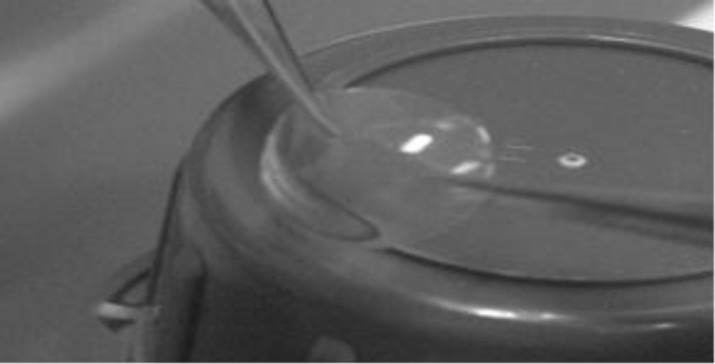
A collagen-based human donor cornea substitute fabricated at the Filatov Institute

Almost transparent BSCS (500µm thick) with single, dotty air bubble inclusions were fabricated based on the described technology (Figure 1). Our BSCS had refractive indices of 1.24-1.3 (human cornea 1.37-1.38). The implants were elastic and strong enough to tolerate well the placement of 12 interrupted stitches during deep lamellar keratoplasty performed on isolated rabbit eyes.

**Figure 2.**
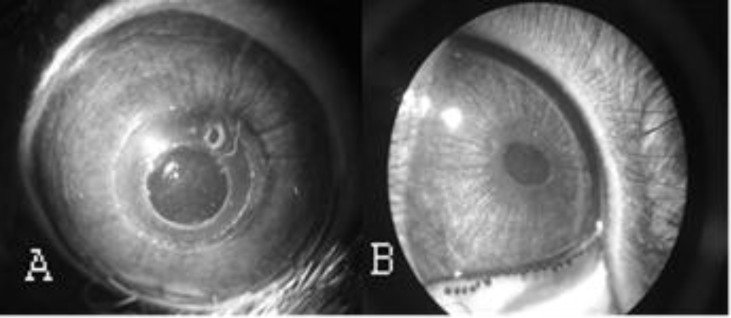
Rabbit cornea in different post-implantation terms, (A) immediately after surgery, (B) 12 months after surgery, where the BSCS is integrated into the host cornea and is visualised because of air bubble inclusions only

**Implantation technique and clinical results**

A new “stitchless” implantation method was performed as follows: a corneal incision 5mm in length and 4/5 thickness in depth was performed with a blade 2mm from the limbus. A corneal “pocket” of 7x7mm was then formed with a spatula through this incision. The BSCS was implanted in the corneal “pocket” with the use of the spatula, and the corneal incision was closed with five 9/0 nylon sutures. Superficial corneal layers over the BSCS were then removed with 5mm trephine (Fig. 2).

No adverse inflammatory reactions were observed in any of the animals. Full epithelial coverage over the BSCS was completed within the first five days post-surgery. Neovascularisation occurred in the stitch placement area only, and resolved itself after the removal of stitches. No neovascularisation was observed in the BSCS location area. When implanted into rabbit corneas, the BSCS remained stably integrated and clear during the 12 month follow-up. Non-intensive opacities within corneal layers (grade 1.5 on a scale of 0 to 4) were observed in 2/8 eyes during the first postoperative week. In one eye the opacity resolved, and a fine opacity (grade 1) remained in the 2nd eye. 

**Histopathological Evaluation**

**Figure 3:**
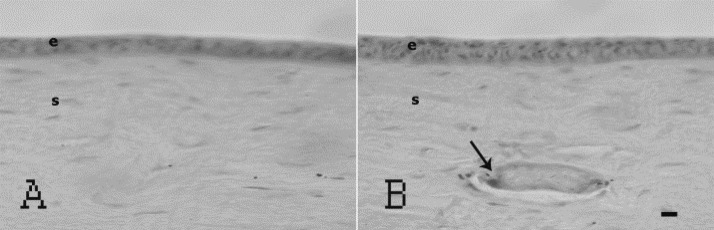
Histology of control and operated corneas at 12 months post-implantation, (A–B): H&E staining of unoperated (A) and operated cornea (B), both showing a stratified epithelium (e) over a stroma (s). A small central piece of still cell-free implant (arrow) lies in reconstituted corneal stroma (B). Scale bar, 50 µm.

Light microscopy confirmed the good integrity of the implants and the absence of inflammation in corneal stroma. The BSCS were covered by corneal epithelium, and the cell-free implants were populated by corneal stromal cells. At 12 months post-operative, only a small piece of cell-free BSCS remained in the central cornea (Fig. 3).

## DISCUSSION

The data of this study confirms the possibility of implant fabrication by the carbodiimide cross-linking of porcine medical grade collagen, as previously described [9] but we had increased the collagen content to approximate the human cornea in optical and mechanical properties. The main difficulty during the fabrication was air bubble elimination from the collagen hydrogel, and this problem is not solved completely. Air bubble inclusions may both decrease the vision of potential recipients and the implant strength. They may also change the refractive index of the latter. It is possible that the implants used had slightly lower refractive indices than both normal human corneas and previously fabricated implants [9, 12, 13]. 

In vivo studies showed that the porcine collagen-based substitutes of corneal stroma were well-tolerated, when implanted into rabbit corneas, where they were seamlessly integrated. The elaborated implantation technique allowed the avoidance of implant degradation and neovascularisation in the stitch placement areas, as previously noticed [9, 12-14]. It is possible that such a technique could prevent a delay in the BSCS epithelial closure, which is a problem found in human studies as a result of the overlying retaining sutures [11]. The tight sutures also appeared to cause irregularities in the surface resulting in astigmatism.

Histological examinations confirmed the good biocompatibility of the implants. At 12 months, there was cell overgrowth but stromal cell in-growth was still incomplete in the central-most areas.

## CONCLUSION

The current data suggest that the carbodiimide crosslinked BSCS fabricated in the Ukraine would most likely be a good alternative to human donor corneas if medical grade porcine collagen is used and the implants are produced under clinical Good Manufacturing practice (cGMP). The new “stitchless” technique of implant retention appears to circumvent the determental effects of implant compression, neovascularisation and prevent delay in the epithelial coverage over the corneal substitutes. Further studies will focus on BSCS quality improvement and comparisons of different BSCS retention techniques. In addition, a phase I clinical study has just started to study the efficacy of the porcine collagen-based BSCS transplantation for persistent corneal ulcer treatment.

## DISCLOSURE

The authors report no conflicts of interest in this work.
